# Genetic Variants of *PICALM* rs541458 Modulate Brain Spontaneous Activity in Older Adults With Amnestic Mild Cognitive Impairment

**DOI:** 10.3389/fneur.2019.00494

**Published:** 2019-05-08

**Authors:** Liying Zhuang, Xiaoyan Liu, Yongmei Shi, Xiaoli Liu, Benyan Luo

**Affiliations:** ^1^Department of Neurology, Zhejiang Hospital, Hangzhou, China; ^2^Department of Neurology and Brain Medical Centre, The First Affiliated Hospital, School of Medicine, Zhejiang University, Hangzhou, China; ^3^Department of Neurology, Affiliated Zhongda Hospital, School of Medicine, Southeast University, Nanjing, China

**Keywords:** amnestic mild cognitive impairment, functional magnetic resonance imaging, amplitude of low frequency fluctuation, *PICALM*, cognition

## Abstract

**Background:** Phosphatidylinositol binding clathrin assembly protein (*PICALM*) rs541458 C allele has been identified and validated to be associated with a reduction of Alzheimer's disease (AD) risk. Nevertheless, the exact mechanisms through which the variant exert its disease-relevant association remain to be elucidated. This study is to determine whether *PICALM* rs541458 polymorphism modulates functional magnetic resonance imaging measured brain spontaneous activity in older adults with amnestic mild cognitive impairment (aMCI).

**Methods:** Thirty five aMCI patients and twenty six healthy controls (HC) were enrolled in this study. Each individual was genotyped for rs541458 and scanned with resting-state functional magnetic resonance imaging. Each group was divided into two subgroups (C carriers and TT genotype). Brain activity was measured with amplitude of low-frequency fluctuation (ALFF).

**Results:** The aMCI patients showed decreased ALFF in left inferior frontal gyrus, superior temporal gyrus and insula, while increased ALFF in right cuneus, calcarine, and bilateral posterior cingulate and precuneus. A significant interaction between diagnosis (aMCI vs. HC) and *PICALM* rs541458 genotype (C carriers vs. TT) on ALFF was observed mainly in the right frontal lobe, with aMCI C carriers and TT genotype in HC showing significantly lower ALFF than HC C carriers. While only negative correlation between ALFF and verbal fluency test was found in HC C carriers (*r* = −0.543, *p* = 0.030).

**Conclusions:** This study provided preliminary evidences that *PICALM* rs541458 variations may modulate the spontaneous brain activity in aMCI patients.

## Introduction

Mild cognitive impairment (MCI) is considered to be a transitional state between normal aging and dementia. Amnestic mild cognitive impairment (aMCI), characterized by predominant episodic memory deficits, has a 10–15% annual risk of conversion to Alzheimer's disease (AD) (1). AD has a substantial genetic component and the heritability is estimated to be around 70% (2). Recent advances in high throughput genomic sequencing technologies have been successful for providing important insights into the genetic underpinnings of AD. Previous large-scale genome wide association studies (GWAS) have identified and validated several novel AD genetic risk loci involved in membrane trafficking, highlighting the importance of endocytosis pathway in AD pathogenesis (3–6). Phosphatidylinositol binding clathrin assembly protein (*PICALM*) is one of those top candidate genes. *PICALM* is located on chromosome 11q14, encoding clathrin assembly lymphoid myeloid leukemia protein (CALM), which plays a key role in clathrin-mediated endocytosis (7, 8). The single nucleotide polymorphism (SNP) rs541458 is 8 kb 5′ to the *PICALM*, with the minor allele C associated with reduced AD risk (3, 4). This kind of association was further replicated in European and Colombian populations (9, 10). Using cerebrospinal fluid (CSF) biomarkers amyloid β (Aβ)-42 and phosphorylated tau (p-tau) as quantitative traits detected significance for *PICALM* rs541458, further providing support for the GWAS findings (11). However, the functional effect of this SNP remains to be determined.

Imaging genetics is an emerging field that aims to identify the associations between genetic variants and quantitative traits extracted from structural or functional neuroimaging data (12–14). Thus, it holds great promise for us to understand the functional role of genes in determining the pathophysiological mechanisms of neuropsychiatric disorders. Multimodal magnetic resonance imaging (MRI) is one of the most frequently neuroimaging techniques incorporated in imaging genetics studies. Previous GWAS of AD-related brain regions in data sets from Alzheimer's disease Neuroimaging Initiative (ADNI) with AD, MCI and controls revealed that *PICALM* was significantly associated with hippocampal volume (15), and entorhinal cortical thickness (15, 16). *PICALM* rs541458 was found to have an effect on cognitive performance, brain structure and resting-state functional magnetic resonance imaging (rs-fMRI) functional connectivity in non-demented elderly (17). Biswal and his colleagues firstly demonstrated that spontaneous blood oxygen level-dependent (BOLD) low frequency (0.01–0.08 Hz) fluctuations in rs-fMRI were physiologically meaningful and were closely related to spontaneous neural activities in the brain (18). Amplitude of low-frequency fluctuation (ALFF) was introduced to measure local BOLD signal variation quantitatively due to regional spontaneous brain activity, based on voxel-wise analysis in the whole brain (19). ALFF was found to be useful and reliable in characterizing the spontaneous brain activity in patients with aMCI or AD (20, 21). However, it remains largely unknown whether genetic variants of *PICALM* rs541458 are associated with the ALFF in the brain.

To our knowledge, this is the first study to address whether *PICALM* rs541458 modulates the spontaneous brain activity in older adults with aMCI. We hypothesized that *PICALM* rs541458 may modulate the ALFF in aMCI patients, and the aims of this study were as follows: (i) to determine whether the ALFF in aMCI was modulated by *PICALM* rs541458 and (ii) whether there is an association between the effects of *PICALM* rs541458 on the ALFF and cognitive function.

## Materials and Methods

### Study Participants

In all, 35 aMCI patients and 26 healthy controls (HC) were recruited from the Affiliated Zhongda Hospital of Southeast University and communities in Nanjing, China. The study was approved by the Southeast University Ethics committee and informed consent was obtained from all participants.

All participants underwent a standardized clinical interview, including demographic inventory, medical history, neurological and mental status. General cognitive function was assessed by the Mini-Mental State Examination (MMSE). A neuropsychological battery test covering episodic memory, attention, visuospatial function, executive domains and language function was utilized. It incorporated auditory verbal learning test (AVLT) 20-min delayed recall, Rey-Osterrieth complex figure test (CFT) 20-min delayed recall, symbol digit modalities test (SDMT), digit span test (DST), clock drawing test (CDT), trail making test (TMT)-B and verbal fluency test (VFT).

The diagnosis of aMCI was made following the recommendations of Petersen et al. (22) and others (23), including (1) a subjective memory complaint, preferably corroborated by an informant, (2) an objective memory impairment, such as a score of less than or equal to 1.5 SD of age adjusted and education adjusted norms on the 20-min delayed recall of AVLT (the cutoff was ≤ 4 correct response on 12 items for ≥8 years of education), (3) MMSE score of 24 or higher, (4) a clinical dementia rating scale (CDR) of 0.5, with at least a 0.5 in the memory domain, (5) normal or minimal impairment in the activities of daily living (ADL), a score of 20 to 26, and (6) absence of dementia, or not sufficient to meet the National Institute of Neurological and Communicative Disorders and Stroke and the Alzheimer's Disease and Related Disorders Association (NINCDS-ADRDA) criteria for AD. HC with normal cognition were required to have a CDR of 0, an MMSE score ≥26, and a delayed recall of AVLT score >4 for ≥8 years of education.

Participants were excluded if they had a history of known stroke, alcoholism, head trauma, Parkinson's disease, epilepsy, major depression or other neurological or psychiatric illness, major medical illness (for example, cancer, anemia, thyroid dysfunction), or severe visual or hearing loss.

### DNA Isolation and SNP Genotyping

Genomic DNA was isolated using the Wizard Genomic DNA purification kit (Promega, Madison, WI, USA) from 4-ml peripheral blood samples according to the manufacturer's protocol. Genotyping of *PICALM* rs541458 and APOE was performed using the iPLEX Assay (SEQUENOM iPLEX® Gold Reagent Kit) according to the assay instructions for PCR amplification, SAP treatment, adjusting extension primers, iPLEX reaction, resin extraction, addition to SpectroCHIP bioarray, and matrix-assisted laser desorption ionization time-of-flight mass spectrometry analysis. Details were described previously (24).

### MRI Data Acquisition

MRI images were acquired in a General Electric 1.5 Tesla scanner (General Electric Medical Systems, Miwaukee, WI, USA) with a homogeneous birdcage head coil. The participants lay supine with their heads snugly fixed by a belt and pads were used to minimize head motion. Resting-state functional images (T2^*^ weighted images) were obtained by gradient-recalled echo-planar imaging (GRE-EPI) sequence: number of slices = 30, thickness = 4.0 mm, gap = 0 mm, in-plane resolution = 3.75 × 3.75 mm^2^, TR = 3,000 ms, TE = 40 ms, flip angle = 90°, acquisition matrix = 64 × 64, FOV = 240 × 240 mm. This acquisition sequence generated 142 volumes in 7 min and 6 s. In addition, three-dimensional T1-weighted axial images covering the whole brain were obtained using a spoiled gradient echo (SPGR) sequence: TR = 9.9 ms, TE = 2.1 ms, thickness = 2.0 mm, gap = 0 mm, flip angle = 15°, FOV = 240 × 240 mm, acquisition matrix = 256 × 192. Participants were instructed to keep their eyes closed, bodies aplanatic and not to think systematically or fall asleep during the scanning.

### Image Preprocessing

Data preprocessing were carried out using DPARSF (25) (http://www.restfmri.net) and Rest (26) (http://www.restfmri.net). The first eight functional volumes were discarded for scanner stabilization and participants' adaption to the circumstances, as in previous studies (27–29). The remaining images were corrected for timing differences and motion effects. No translation or rotation parameters of head motion in any given data set exceeded ±3 mm or ±3°. Next, the individual structural images (T1-weighted SPGR images) were co-registered to the mean functional image after motion correction using a linear transformation. The transformed structural images were then segmented into gray matter, white matter and cerebrospinal fluid using a unified segmentation algorithm. The motion corrected functional volumes were spatially normalized to the Montreal Neurological Institute space and resampled to 3 × 3 × 3 mm^3^ voxels using the normalization parameters estimated during the unified segmentation. The resulting images were smoothed with an isotropic Gaussian kernel with a FWHM of 6 mm. Then, the linear trend of time courses was removed.

### ALFF Calculation

ALFF was calculated using REST software similar to that used in previous studies (19, 30). Firstly, the time series of each given voxel was converted to the frequency domain using a Fast Fourier Transform and the power spectrum was then acquired. Secondly, the square root was computed at each frequency of the power spectrum and averaged between 0.01 and 0.08 Hz, and this averaged square root was termed ALFF. Finally, for standardization, the value of ALFF of each voxel was divided by the global mean ALFF value within a whole-brain mask.

### Voxel-Wise-Based Gray Matter Volume Correction

The voxel-wise gray matter volumes were included as covariates in the ALFF analysis to control for possible difference in ALFF due to anatomical variation (31). Firstly, individual gray matter volume maps were obtained using voxel-based morphometry (VBM). Secondly, the maps were transformed into the same standard space as the resting-state fMRI imaging using affine linear registration. Finally, these resulting voxel-wise gray matter volume maps were input as covariates in the analysis of the functional data. The voxel-wise-based gray matter volume correction was performed for each participant.

### Statistical Analysis

Haploview 4.0 was applied to analyze the Hardy-Weinberg equilibrium and minor allele frequency of *PICALM* rs541458, determining the SNP with a Hardy-Weinberg *P*-value > 0.001 and minor allele frequency >0.05. Demographics and neuropsychological data analysis were performed using two-way analysis of variance (ANOVA) for continuous variables and using chi-square test for categorical variables. Specially, the main effects of diagnosis (aMCI vs. HC) and *PICALM* rs541458 genotype (C carriers vs. TT genotype), and diagnosis-by-genotype interactions were assessed. These analyses were implemented in SPSS 17.0, and the statistical significance was set at *P* < 0.05.

A voxel-wise two-way ANOVA was performed to analyze the main effects of diagnosis (aMCI vs. HC) and *PICALM* rs541458 genotype (C carriers vs. TT genotype), and the diagnosis-by-genotype interactions on ALFF maps using SPM12 (http://www.fil.ion.ucl.ac.uk/spm). *Post-hoc t*-tests were performed to explore the details of those clusters showing significant main effects and interactions. All the statistical maps were corrected for multiple comparisons according to the AlphaSim program based on Monte Carlo simulation algorithm (α = 0.05, voxel-wise *P* < 0.05, cluster sizes >4,941 mm^3^; http://afni.nimh.nih.gov/pub/dist/doc/manual/AlphaSim.pdf). Finally, a correlative analysis was performed between the neuropsychological test scores and the ALFF values of the clusters showing significant interactions (*P* < 0.05).

## Results

### Demographic and Neuropsychological Results

Table 1 illustrates the demographic and neuropsychological data for aMCI and HC participants stratified by *PICALM* rs541458 genotype. The four subgroups did not differ in age, gender, education and APOEε4 status (all *P*-values > 0.05). Two-way ANOVA analyses revealed the main effects of diagnosis and *PICALM* rs541458 genotype, and the diagnosis-by-genotype interactions on neuropsychological tests. Briefly, a significant main effect of diagnosis on each cognitive domain was observed, with the aMCI group showing worse cognitive performance in episodic memory (AVLT-delayed recall and CFT-delayed recall), attention (SDMT and DST), executive (TMT-B), visuospatial (CDT) and language (VFT) function than the control group (all *P*-values < 0.05). There was no significant main effect of *PICALM* rs541458 genotype on any cognitive measure (all *P*-values > 0.05). Further, we did not observe any significant interaction between diagnosis and *PICALM* rs541458 genotype on those cognitive measures (all *P*-values > 0.05). Further analysis showed no difference between genotypes in the aMCI and control group (see Table S1).

**Table 1:** Demographic and neuropsychological result.

	**aMCI group (*N* = 35)**		**control group (*N* = 26)**		***P*-values**		
	***PICALM* C carriers (*N* = 24)**	***PICALM* TT (*N* = 11)**	***PICALM* C carriers (*N* = 16)**	***PICALM* TT (*N* = 10)**	**Diagnosis**	**Genotype**	**Interaction**
Age (years)	70.42 ± 4.39	71.91 ± 4.39	70.06 ± 6.58	67.80 ± 2.39	0.100	0.774	0.165
Education (years)	14.08 ± 2.83	13.18 ± 3.37	14.38 ± 2.60	15.75 ± 2.86	0.073	0.763	0.151
Gender (male/female)	16/8	7/4	11/5	5/5	0.667	0.419	0.559
APOEε4 (yes/no)	9/15	1/10	0/16	1/9	0.067	0.352	0.055
MMSE	27.04 ± 1.49	27.64 ± 1.36	28.31 ± 1.40	27.90 ± 1.29	0.050	0.813	0.195
AVLT-delayed recall	2.63 ± 1.31	3.36 ± 1.50	8.13 ± 1.59	8.80 ± 1.69	<0.001^*^	0.085	0.937
CFT-delayed recall	11.13 ± 7.64	13.18 ± 8.15	19.40 ± 5.71	15.40 ± 7.34	0.011^*^	0.626	0.133
SDMT	27.58 ± 9.27	29.09 ± 12.00	33.94 ± 10.36	36.60 ± 5.54	0.011^*^	0.429	0.826
DST	12.13 ± 2.09	12.09 ± 1.81	13.25 ± 1.57	13.20 ± 2.57	0.045^*^	0.939	0.988
TMT-B (seconds)	197.50 ± 84.70	155.82 ± 63.18	135.56 ± 40.83	135.50 ± 27.45	0.022^*^	0.238	0.239
CDT	8.33 ± 1.34	7.91 ± 2.55	9.20 ± 0.68	8.80 ± 1.48	0.041^*^	0.331	0.977
VFT	10.46 ± 2.93	10.18 ± 1.47	13.00 ± 2.99	13.70 ± 2.31	<0.001^*^	0.770	0.501

*Data were presented as the mean ± standard deviation (SD). P-values were obtained by two way analysis of variance (ANOVA)*.

* *indicates a statistical difference between groups, P < 0.05. MMSE, Mini-Mental State examination; AVLT, Auditory Verbal Learning Test; CFT, Rey-Osterrieth Complex Figure Test; SDMT, Symbol Digit Modalities Test; DST, Digit Span Test; TMT, Trail Making Test; CDT, Clock Drawing Test; VFT, Verbal Fluency Test*.

### The ANOVA Results of ALFF

**Figure 1:**
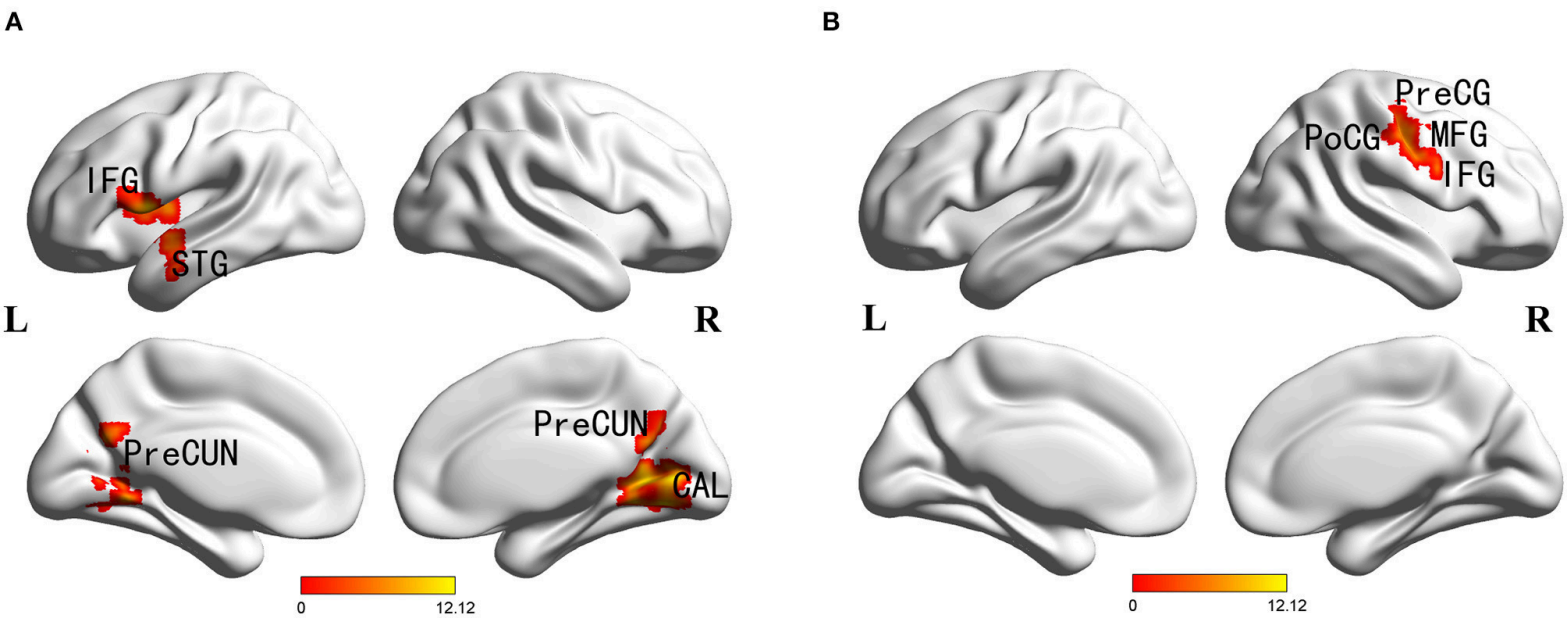
Diagnosis × genotype ANOVA of ALFF. Thresholds were set at a corrected *p* < 0.05, determined by Monte Carlo stimulation. **(A)** Main effect of diagnosis. **(B)** Diagnosis×genotype interaction. IFG, inferior frontal gyrus; STG, superior temporal gyrus; CAL, calcarine; PreCUN, precuneus; PreCG, precentral gyrus; PoCG, postcentral gyrus; MFG, middle frontal gyrus.

Main effects of diagnosis were identified in the frontal cortex (left inferior frontal gyrus), temporal cortex (left superior temporal gyrus), parietal cortex (bilateral precuneus), occipital cortex (right cuneus and calcarine), posterior cingulate and left insula. There were no significant main effects of *PICALM* rs541458 genotype in any brain region. While, significant interactions between the effects of diagnosis and genotype on ALFF were found in frontal cortex (right precentral gyrus, middle and inferior frontal gyrus) and parietal cortex (right postcentral gyrus) (for details see Table 2 and Figure 1).

**Table 2:** Diagnosis × genotype ANOVA of ALFF.

**Brain region**	**BA**	**Peak MNI coordinates (mm)**			**Peak *F*-value**	**Cluster size**
		***x***	***y***	***z***		
**MAIN EFFECT OF DIAGNOSIS**					
R cuneus/R calcarine/B posterior cingulate/B precuneus	30/18	9	−72	6	12.12	28,431
L inferior frontal gyrus/L insula/L superior temporal gyrus	44	−54	6	9	10.56	5,400
**MAIN EFFECT OF GENOTYPE**					
None						
**DIAGNOSIS × GENOTYPE INTERACTION**					
R precentral gyrus/R postcentral gyrus/R middle frontal gyrus/R inferior frontal gyrus	6/4	39	−6	9	10.49	11,421

*All the regions survived the Monte Carlo stimulations, and the thresholds were set as voxel-wise P < 0.05, cluster size larger than 4,941 mm^3^. R, right; L, left; B, bilateral; BA, Brodmann's area*.

### *Post-hoc* Analyses

**Figure 2:**
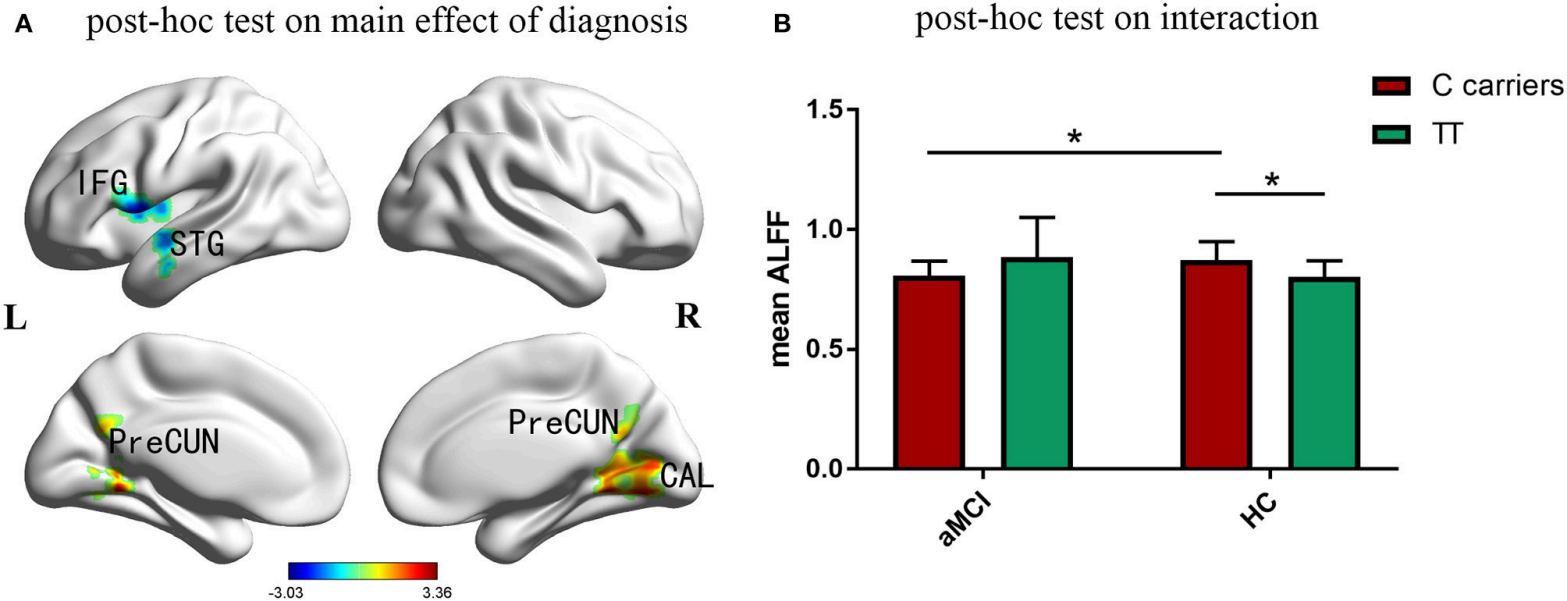
*Post-hoc* analyses. Thresholds were set at a corrected *p* < 0.05, determined by Monte Carlo stimulation. **(A)** Compared with the control group, aMCI participants showed a significant decrease of ALFF in left inferior frontal gyrus, superior temporal gyrus and insula, while increased ALFF in right cuneus, calcarine and bilateral posterior cingulate and precuneus. **(B)** Further *post-hoc* tests of the significant interactions of diagnosis and genotype revealed that, aMCI C carriers showed decreased ALFF in the interactive brain regions compared with control C carriers. In addition, the ALFF value of C carriers was higher than that of TT genotype in control group. IFG inferior frontal gyrus, STG superior temporal gyrus, CAL calcarine, PreCUN precuneus. *Indicates a statistical difference between groups, *P* < 0.05.

Compared with the control group, aMCI participants showed a significant decrease of ALFF in left inferior frontal gyrus, superior temporal gyrus and insula, while increased ALFF in right cuneus, calcarine and bilateral posterior cingulate and precuneus (Figure 2A). Figure 2B indicated that the effects of *PIACLM* genotype on ALFF were visually opposite in aMCI and control group. While further *post-hoc* tests of the significant interactions of diagnosis and genotype revealed that, aMCI C carriers showed decreased ALFF in the interactive brain regions compared with control C carriers. In addition, the ALFF value of C carriers was higher than that of TT genotype in control group.

### Behavioral Significance of ALFF in Regions Associated With ANOVA Interactions

**Figure 3:**
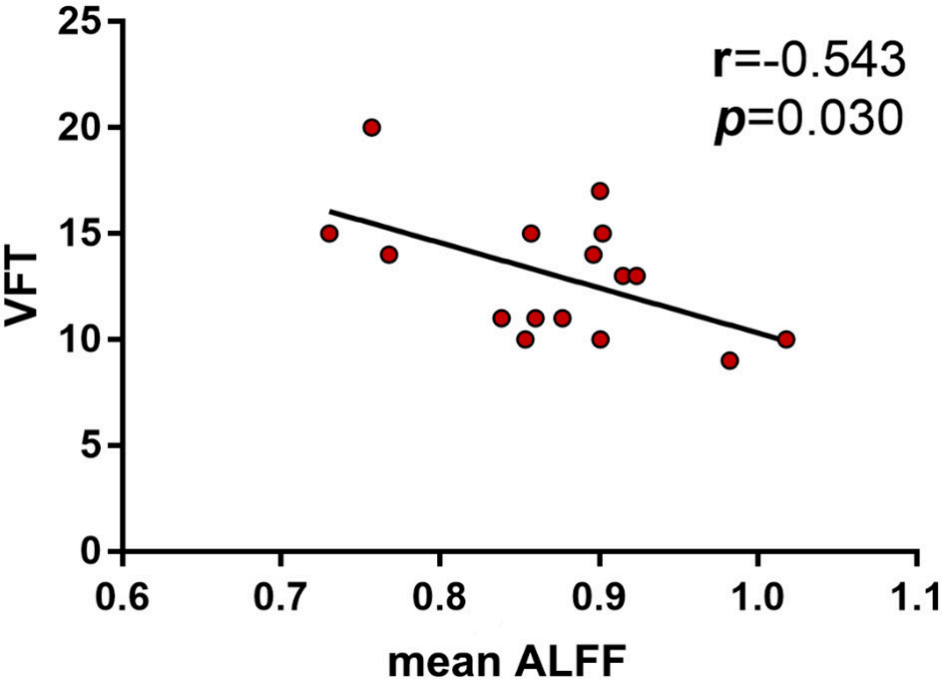
Correlations between ALFF and neuropsychological tests showed the mean ALFF values of the interactive brain regions were negatively correlated with the language function (VFT) in HC *PICALM* C carriers (*r* = −0.543, *P* = 0.030).

We were particularly interested in the behavioral significance of ALFF in regions associated with ANOVA interactions. Therefore, the ALFF of these regions from each participant was analyzed. We found that the mean ALFF values of these regions were negatively correlated with the language function (VFT) in HC *PICALM* C carriers (*r* = −0.543, *P* = 0.030) (Figure 3). While, there were no significant correlations between the mean values of ALFF and any cognitive measure in the other three subgroups.

## Discussion

This was the first study to investigate the potential effect of *PICALM* rs541458 polymorphism on brain regional spontaneous activity in aMCI patients. *PICALM* is located on chromosome 11q14 and predominantly expressed in neurons (32). The SNP rs541458 has been shown to be in linkage disequilibrium with rs3851179, which is located 88.5kb 5′ to the *PICALM* gene (3). It is possible that the presence of both SNPs in the 5′ region outside the gene may have an effect on the expression of *PICALM*. The gene product CALM co-localizes with amyloid precursor protein (APP) *in vitro* and *in vivo*, regulating APP internalization and subsequent Aβ generation, thus contributing to amyloid plaque load by its influence on Aβ metabolism (32, 33). CALM has also been reported to contribute to AD development by modulating autophagy and the clearance of tau protein, which is an autophagy substrate and causatively linked to AD (34). Another work has suggested that CALM is associated with the development of AD tau pathology (35). The genetic variants of rs541458 in *PICALM* was shown to have an effect on CSF-Aβ42 levels, with the most significant decrease in CSF-Aβ42 in homozygous carriers of the T allele (36).

The ANOVA on neuropsychological data revealed main effects of diagnosis, with deficits in episodic memory, attention, executive, visuospatial and language functions of aMCI patients. However, we did not find significant main effect of *PICALM* rs541458 nor any interaction between diagnosis and *PICALM* genotype on each cognitive measure. The effect of *PICALM* rs541458 was not significant for all neuropsychological tests in non-demented elderly, while an interactive effects of rs541458 and age existed with regard to executive function and processing speed previously (17). While another work observed a cumulative effect of *PICALM* rs541458 and some other top genetic polymorphisms on episodic memory (37). The possible explanation for the lack of genotype effect on cognition could be the small sample size analyzed in our study.

In subsequent analyses of ALFF, there was a main effect of diagnosis, but not of *PICALM* rs541458 genotype. The current study replicated the alterations of ALFF in aMCI patients as our previous findings (24), including decreased ALFF in left inferior frontal gyrus and superior temporal gysus, while increased ALFF in precuneus and right calcarine. Other studies also demonstrated decreased ALFF in left inferior frontal gyrus (38) and superior temporal gyrus (39) in MCI patients. A recent study employed the regional homogeneity (ReHo) measure to explore the characteristics of local brain activity and found decreased ReHo index in the left superior temporal gyrus in MCI patients (40) In addition, we found increased ALFF in posterior cingulate and right cuneus, besides precuneus and right calcarine. Our findings shared some overlap with the default mode network (DMN) of MCI and AD. The increased ALFF in posterior cingulate and precuneus were in contradiction with some previous studies, which showed a decrease of ALFF (21, 41, 42). Nevertheless, other studies reported the similar findings as us with different kind of approaches. AD patients showed higher ReHo in precuneus cortex than in matched healthy controls previously, which was thought to be a reflection of compensatory brain responses (43). Another study reported that the changes of DMN functional connectivity were non-linear throughout the course of aMCI, and there was an increase in DMN functional connectivity from mild aMCI to moderate aMCI, then a decrease to severe aMCI (44). One longitudinal study observed posterior cingulate and precuneus hyper-functional connectivity at baseline in aMCI subjects, while a substantial decrease of the connections was evident after 20 months follow up (45). So the possibility for these inconsistent results regarding to those brain regions, especially posterior cingulate and precuneus may exist in the compensatory mechanisms reported in AD (46).

Moreover, our current study found an interaction between diagnosis and *PICALM* rs541458 genotype on ALFF mainly in right frontal lobe (precentral gyrus, middle and inferior frontal gyrus). The expression of *PICALM* was reported to be significantly increased in the frontal cortex of AD brain tissue before (47), and mass spectrometry quantification of CALM in human frontal cortex has been carried out, with a consensus value of about 0.62 pmol/mg tissue protein (48). Further *post-hoc* analysis demonstrated C allele carriers in HC showed higher ALFF than TT genotype, and C carriers in HC showed higher ALFF than their counterparts in aMCI group. These findings support our previously proposed hypothesis that *PICALM* rs541458 modulates the effect of diagnosis on ALFF, and the protective C allele of *PICALM* rs541458 has a positive effect of regional brain spontaneous activity in primary moter cortex. Similar effect of rs541458 was observed in non-demented elderly, with higher functional connectivity of the left superior parietal gyrus in CC genotype than in the T allele carriers <65 years old group, and with higher gray matter volume of the left middle temporal gyrus in CC genotype than in the T allele carriers ≥65 years old group (17). Interestingly, it appeared that the ALFF value in each aMCI subgroup was in an opposite trend with that in HC, although without significance. Furthermore, we observed a negative correlation between VFT and ALFF with regard to the interactive effects of *PICALM* rs541458 and diagnosis in HC C carriers, while not in aMCI group. *PICALM* rs541458 C allele distribution was found significantly different in AD patients and cognitive normal subjects, with a decrease of C allele with increasing age only in the AD group, while remaining constantly distributed in all age strata of cognitive normal subjects (49). A different role of *PICALM* rs541458 in healthy and impaired cognitive aging was proposed, and age may modulate the effects of *PICALM* rs541458 on cognitive performance and brain function. In this study, we did not split the cohort into different age groups as there were no subjects with an age lower than 65 years old in aMCI group. Further studies are needed to explore whether this different role of *PICALM* rs541458 on ALFF in aMCI and healthy control were modulated by age in larger samples.

There are several biological and technical limitations in our study. First, since the aMCI and healthy control subjects were diagnosed clinically without consideration of relevant disease associated biomarkers, there may exist some concerns about disease heterogeneity and latent disease in control individuals. Thus, using enrollment criteria combining clinical status and specific biomarkers of AD as refined by National Institute on Aging in future will improve the likelihood of scientific discovery and add some biological context to the results (50, 51). Second, the sample size was small, individuals with CC and CT genotype were then integrated into one subgroup. As a result, no significant main effect of genotype was observed, which could be further explored in a larger sample study. Finally, given the cross-sectional nature of this study, we cannot draw conclusions regarding causal relationships. Further analysis of *PICALM* rs541458 effect on spontaneous brain activity using a longitudinal design is what we plan to do next.

In conclusion, this is the first study to assess the gene-imaging-behavior associations involving *PICALM* rs541458 in aMCI participants. Besides altered ALFF in inferior frontal gyrus, superior temporal gyrus, insula, calcarine, precuneus, cuneus and PCC in aMCI, this study provided preliminary evidences that *PICALM* rs541458 may moderate the spontaneous brain activity in older adults with aMCI.

## Ethics Statement

This study was carried out in accordance with the recommendations of Southeast University of guidelines, Southeast University of committee with written informed consent from all subjects. All subjects gave written informed consent in accordance with the Declaration of Helsinki. The protocol was approved by the Southeast University of committee.

## Author Contributions

LZ analyzed the data, made pictures and tables and wrote the paper. XYL helped analyze the data. YS contributed to the conception, design and acquisition data. XLL and BL contributed to the critical review and revision of the manuscript. All authors reviewed and approved the manuscript.

### Conflict of Interest Statement

The authors declare that the research was conducted in the absence of any commercial or financial relationships that could be construed as a potential conflict of interest.
