# Supplementary material for: Genetic Variants of PICALM rs541458 Modulate Brain Spontaneous Activity in Older Adults With Amnestic Mild Cognitive Impairment
Source: Front Neurol. 2019 May 8;10:494. doi: 10.3389/fneur.2019.00494 (PMC6517502; doi:10.3389/fneur.2019.00494)
Supplement: Supplementary file 1 [file Table_1.DOCX]

# Supplementary Tables

**Table S1**. Demographic and neuropsychological results between genotype in aMCI and control groups

|  | **aMCI group** (N=35) | |  |  | **control group** (N=26) | |  |
| --- | --- | --- | --- | --- | --- | --- | --- |
|  | ***PICALM* C carriers** (N=24) | ***PICALM* TT** (N=11) | ***P*** |  | ***PICALM* C carriers** (N=16) | ***PICALM* TT** (N=10) | ***P*** |
| Age (years) | 70.42±4.39 | 71.91±4.39 | 0.371 |  | 70.06±6.58 | 67.80±2.39 | 0.396 |
| Education (years) | 14.08±2.83 | 13.18±3.37 | 0.389 |  | 14.38±2.60 | 15.75±2.86 | 0.073 |
| Gender (male/female) | 16∕8 | 7∕4 | 1.000 |  | 11∕5 | 5∕5 | 0.425 |
| APOEε4 (yes/no) | 9∕15 | 1∕10 | 0.120 |  | 0∕16 | 1∕9 | 0.385 |
| MMSE | 27.04±1.49 | 27.64±1.36 | 0.294 |  | 28.31±1.40 | 27.90±1.29 | 0.362 |
| AVLT-delayed recall | 2.63±1.31 | 3.36±1.50 | 0.110 |  | 8.13±1.59 | 8.80±1.69 | 0.313 |
| CFT-delayed recall | 11.13±7.64 | 13.18±8.15 | 0.474 |  | 19.40±5.71 | 15.40±7.34 | 0.220 |
| SDMT | 27.58±9.27 | 29.09±12.00 | 0.687 |  | 33.94±10.36 | 36.60±5.54 | 0.464 |
| DST | 12.13±2.09 | 12.09±1.81 | 0.963 |  | 13.25±1.57 | 13.20±2.57 | 0.951 |
| TMT-B (seconds) | 197.50±84.70 | 155.82±63.18 | 0.160 |  | 135.56±40.83 | 135.50±27.45 | 0.997 |
| CDT | 8.33±1.34 | 7.91±2.55 | 0.838 |  | 9.20±0.68 | 8.80±1.48 | 0.692 |
| VFT | 10.46±2.93 | 10.18±1.47 | 0.747 |  | 13.00±2.99 | 13.70±2.31 | 0.534 |

Data were presented as the mean ± standard deviation (SD). T tests or Mann-Whitney U tests were performed for continuous variables, χ2 tests were performed for dichotomous variables. MMSE: Mini-Mental State examination; AVLT: Auditory Verbal Learning Test; CFT: Rey-Osterrieth Complex Figure Test; SDMT: Symbol Digit Modalities Test; DST: Digit Span Test; TMT: Trail Making Test; CDT: Clock Drawing Test; VFT: Verbal Fluency Test
